# Comparative RNA seq analysis of the New Zealand glowworm *Arachnocampa luminosa* reveals bioluminescence-related genes

**DOI:** 10.1186/s12864-015-2006-2

**Published:** 2015-10-21

**Authors:** Miriam L. Sharpe, Peter K. Dearden, Gregory Gimenez, Kurt L. Krause

**Affiliations:** Department of Biochemistry, University of Otago, Dunedin, New Zealand; Otago Genomics & Bioinformatics Facility, University of Otago, Dunedin, New Zealand

## Abstract

**Background:**

The New Zealand glowworm is the larva of a carnivorous fungus gnat that produces bioluminescence to attract prey. The bioluminescent system of the glowworm is evolutionarily distinct from other well-characterised systems, especially that of the fireflies, and the molecules involved have not yet been identified. We have used high throughput sequencing technology to produce a transcriptome for the glowworm and identify transcripts encoding proteins that are likely to be involved in glowworm bioluminescence.

**Results:**

Here we report the sequencing and annotation of the first transcriptome of the glowworm, and a differential analysis of expression from the glowworm light organ compared with non-light organ tissue. The analysis identified six transcripts encoding proteins that are potentially involved in glowworm bioluminescence. Three of these proteins are members of the ANL superfamily of adenylating enzymes, with similar amino acid sequences to that of the luciferase enzyme found in fireflies (31 to 37 % identical), and are candidate luciferases for the glowworm bioluminescent system. The remaining three transcripts encode putative aminoacylase, phosphatidylethanolamine-binding and glutathione S-transferase proteins.

**Conclusions:**

This research provides a basis for further biochemical studies into how the glowworm produces light, and a source of genetic information to aid future ecological and evolutionary studies of the glowworm.

**Electronic supplementary material:**

The online version of this article (doi:10.1186/s12864-015-2006-2) contains supplementary material, which is available to authorized users.

## Background

In caves and forested river gorges across New Zealand, an abundance of star-like lights can be seen when it is dark. The creature responsible for this light, known locally as a glowworm, is the carnivorous larva of a fungus gnat, *Arachnocampa luminosa*. The larva lies in a mucous hammock, hanging long sticky silk threads below it like fishing lines, and luminesces from a small light organ located at the end of its tail. Small flying insects are attracted by the light, become entangled in the sticky lines, and are then consumed by the glowworm [1, 2].

Despite their common name, glowworms are actually a type of fly (order Diptera, family Keroplatidae) [3]. Confusingly, some firefly larvae and adults are also referred to as glowworms, but the well-studied bioluminescent beetles (including fireflies, click beetles and railroad worms) are members of a different order – Coeloptera (superfamily Elateroidea) [4]. Diptera and Coleoptera diverged about 330 million years ago, with no known bioluminescent species intervening [5–7], which indicates that bioluminescence evolved independently in these insects.

The ability to bioluminesce has evolved many times. One recent estimate suggests that bioluminescence has evolved at least 40 times across extant organisms, possibly more than 50 times, when counting the number of distinct light-producing chemical mechanisms across monophyletic lineages [6]. Despite their differences and separate evolutionary origins, all bioluminescent systems that have been studied produce light by oxidation of a light-emitting substrate (generically referred to as a luciferin) catalyzed by an enzyme (a luciferase). Luciferase enzymes have extremely varied structures, mechanisms and substrate specificities [8].

Researchers have studied the biochemistry used by *Arachnocampa* to produce light [9–11], but many details remain elusive, including the identities of the luciferase and luciferin. Although both the glowworm and firefly systems use ATP to bioluminesce, the chemistries of bioluminescence in the two creatures are distinct. When mixed, the substrate for the firefly system (luciferin) and the glowworm luciferase do not produce light [9], which implies that they use different luciferase enzymes and substrates. The physiology of the glowworm light organ is also unique. It is made up of the swollen distal tips of the four Malpighian tubules [12, 13]. Malpighian tubules are part of the insect excretory system, analogous to the vertebrate kidneys, and are not part of any other insect bioluminescence system [14].

The ability of firefly, bacteria (such as *Vibrio harveyi*) and sea pansy (*Renilla*) luciferases and luciferins to produce easily-measured light has led to the use of these systems as tools in biomedical and biological research, for example as genetic reporters, drug screening assays, bioluminescence imaging, and assays for the presence of ATP or calcium [15, 16]. Understanding the molecular basis of light generation in glowworms will not only expand our understanding of how bioluminescence works, but may also lead to novel bioluminescent applications. For example, the glowworm system uses a different luciferin substrate and produces a different bioluminescent spectra maximum than currently used bioluminescent research tools, therefore the glowworm system could be used in conjunction with existing bioluminescent applications, for example, to detect several compounds in one sample or monitor expression of multiple genes simultaneously.

One approach to revealing the molecular physiology of glowworm bioluminescence is to sequence the transcriptome of the organism. Transcriptome sequencing is a relatively cheap and easy way of providing genome-wide sequence data for non-model organisms for which no genome sequence data is available [17, 18].

Sequencing on a genome-wide scale is still a new approach for investigating bioluminescence. Although reported high-throughput sequencing of bioluminescent creatures so far include four genomes (the ctenophore *Mnemiopsis leidyi* [19], various strains of *V. fischeri* [20–22], the luminous mushroom *Mycena chloropho* [23], and the European brittle star *Amphiura filiformis* [24]), and several transcriptomes (the European brittle star [24], cypridinid ostracods or seed shrimp [25], the Oplophorid shrimp [26], the luminous mushroom [23], and the dinoflagellate *Lingulodinium polyedrum* [27, 28]), none of these studies have reported any detailed analyses specifically looking at the differences in transcripts produced between luminous and non-luminous tissues. It should be noted that a limited transcriptional profiling study has been carried out on *A. luminosa* [29]. The authors of this study sequenced 537 cDNAs that were constructed from light organ expressed sequence tags using the Sanger method, and did not include a comparison between tissues. The same research group also carried out a small transcriptional survey on a cDNA library from *Macrolampis* sp2 firefly lanterns [30].

Here we present the first in-depth sequencing of polyadenylated RNAs from the New Zealand glowworm, and a detailed analysis at the transcriptomic level of luminescent versus nonluminescent tissue, through which we have identified six proteins that are likely to be involved in bioluminescence. This research provides a basis for biochemical studies into how the glowworm produces light, and a source of genetic information to aid future ecological and evolutionary studies of the glowworm.

## Results and discussion

### Experimental plan

We carried out two separate transcriptome sequencing experiments, on biological replicates, using the most appropriate bioinformatic processing and analyses for each approach. We first performed 454 GS-FLX sequencing because there was no genomics information available for the species, and then used Illumina sequencing to validate those results, and to provide greater sequencing depth to support a differential gene expression analysis. RNA was extracted from the light organ and from the rest of the body (non-light organ). The two experiments differed in the use of biological replicates, sequencing platform, and analysis software (Additional file 1: Figure S1).

### Sequencing, read cleaning and *de novo* assembly

#### 454

**Fig. 1:**
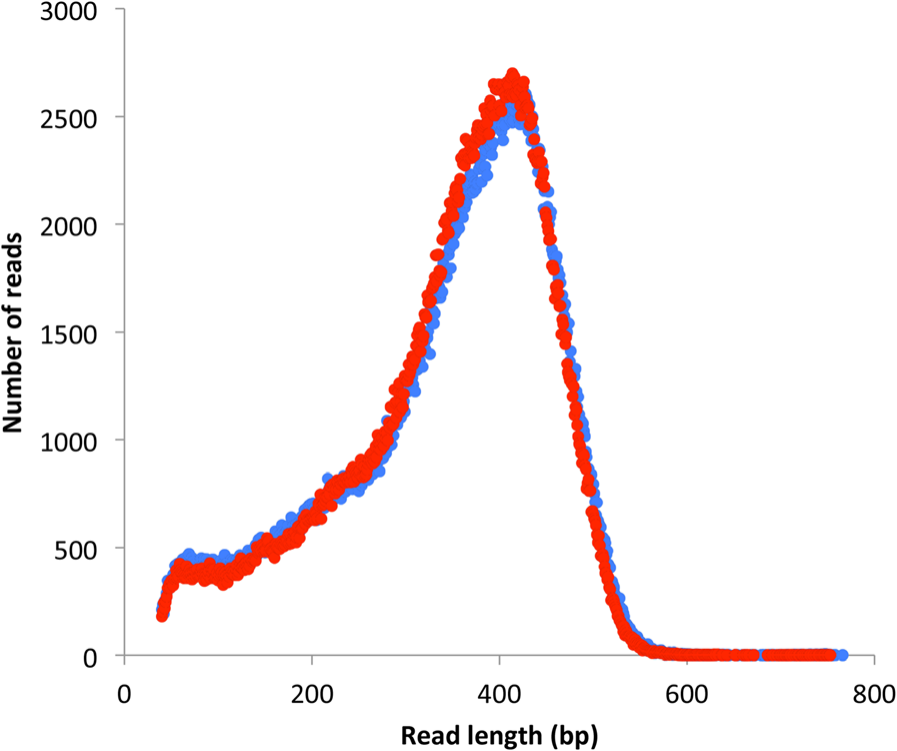
Distribution of read lengths from 454 sequencing of light organ (blue) and non-light organ (red) cDNA libraries

mRNA was isolated from two samples: one prepared from non-light organ tissue (~200 mg of tissue from eight glowworms) and one prepared from light organ tissue (~420 mg of tissue from 172 glowworms). After cDNA synthesis, 454 GS-FLX sequencing for each library was carried out on one-half of a pico-titer plate. A total of about 1.12 million high quality reads were obtained, amounting to almost 400 Mbp (Table 1; Fig. 1). Reads, including singletons, were merged from both libraries for *de novo* transcript assembly, which was carried out using CLC Genomics Workbench 5.1, and yielded a reference transcriptome of 18,794 transcripts, with an N50 of 897 (Table 2).

**Table 1:** Summary statistics for reads from 454 GS-FLX sequencing

	Total HQ reads	Total HQ sequence (bp)	Average read length	% Mixed	% Dots
Light organ tissue	559 773	192 760 671	344	16.13	11.25
Non-light organ tissue	564 835	194 425 594	344	12.07	11.37
Combined	1 124 608	387 186 265	344	14.09	11.31

HQ = high quality; % Mixed = percentage of reads filtered out by the mixed filter, where a mixed read is the result of simultaneously sequencing a mixture of different DNA molecules; % Dots = percentage of reads filtered by the dots filter, where a dot is an instance of three successive nucleotide flows that record no incorporation

**Table 2:** *De novo* assembly statistics

Input	Assembly software	Number of transcripts	Number of bases assembled	N50 (bp)	Number of singleton reads	Median contig length (bp)	Mean contig length (bp)	Maximum contig length (bp)
454 reads (two libraries merged)	CLC Genomics Workbench	18 794	14 257 486	897	94 753	586	759	11 217
Illumina reads (six libraries merged)	Trinity	196 766	187 289 921	1 828	NA	447	952	30 278

N50 size = the length such that 50 % of the assembled genome lies in N50 size or greater; NA = not applicable to results from this assembly program

#### Illumina

We sequenced six cDNA libraries using the Illumina HiSeq-2000 sequencer, each prepared from either the light organ or non-light organ mRNA of three individual glowworms. The increased number of biological replicates in the second experiment provided it with increased statistical power for the subsequent RNA-seq analysis [31]. The Illumina platform also provided us with information on strand origin, i.e. from which of the two DNA strands a given RNA transcript was derived. This information can increase the percentage of alignable reads, thereby improving transcript reconstruction compared with non-strand specific data of known strand origin [32].

**Fig. 2:**
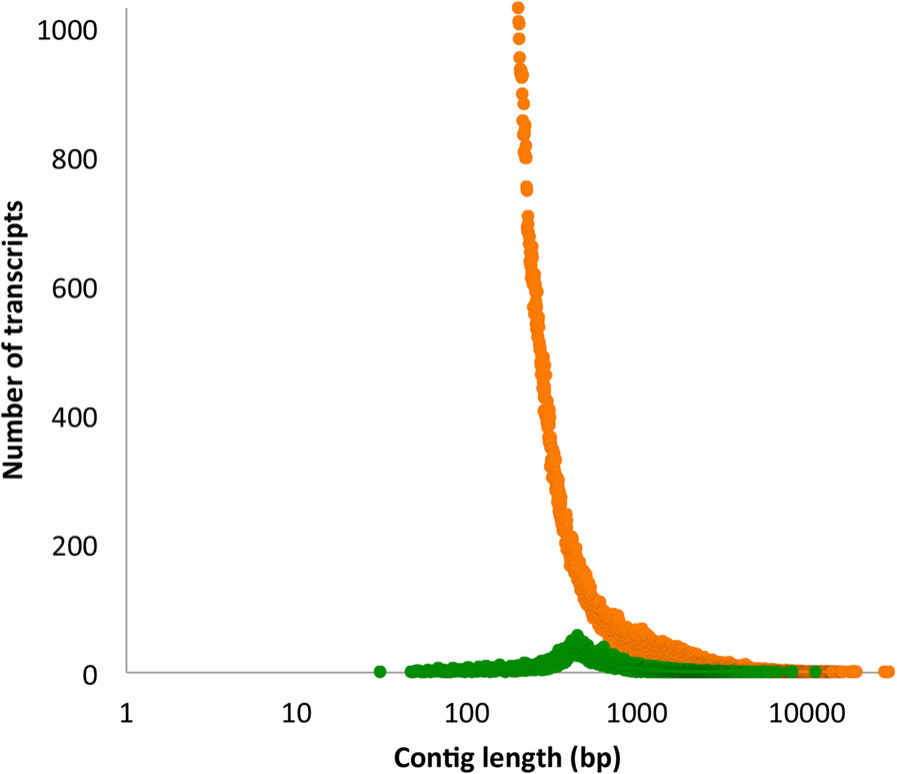
Distribution of contig lengths for 454/CLC Genomics Workbench (green) and Illumina/Trinity (orange) assemblies

The Illumina machine generated 37.1 to 44.7 million pairs of 200 base length paired-end reads for each library (Table 3). In order to improve the quality of the data, we removed adapter sequences, trimmed low quality bases (Q < 20) from both ends of reads and discarded reads less than 50 bases in length. The resulting 29.5 to 35.7 million high quality reads per library (79 to 80 % of total raw reads) were merged together for *de novo* transcript assembly using the Trinity package, producing a *de novo* assembly containing 187,289,921 bases and a total of 196,766 transcripts (Table 2). A graph of the contig length distribution highlights the differences in contig size and number between the two assemblies (Fig. 2).

**Table 3:** Summary statistics for reads from Illumina sequencing

Sample	Raw reads	Trimmed, quality filtered reads	Trimmed, quality filtered reads (% of raw reads)
Glowworm 1 light organ	44 776 272	35 671 872	80 %
Glowworm 1 non-light organ	39 450 268	31 259 202	79 %
Glowworm 2 light organ	41 122 414	32 769 962	80 %
Glowworm 2 non-light organ	37 092 452	29 526 444	80 %
Glowworm 3 light organ	40 295 844	32 042 384	80 %
Glowworm 3 non-light organ	39 697 568	31 624 498	80 %
Combined	242 434 818	192 894 362	80 %

### Functional annotation and gene ontology of the glowworm transcriptome

**Fig. 3:**
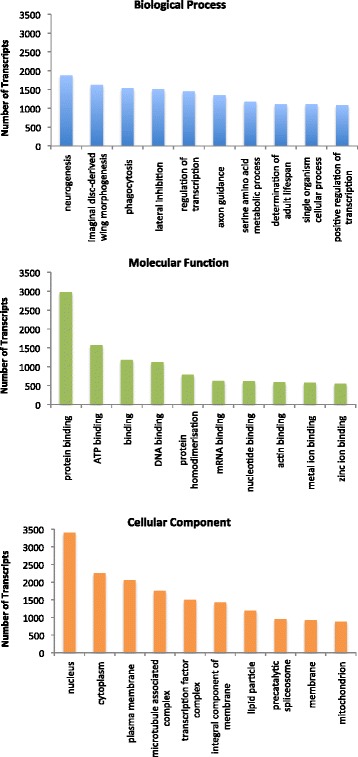
Gene ontology annotations. Pictured are the top ten GO terms for each of the three GO categories

In order to provide descriptions of the functions and properties of as many glowworm gene products as possible, BLASTX searches were performed for each transcript above 1 kb in size from the Illumina/Trinity transcriptome assembly against the *Drosophila* RefSeq non-redundant database at the National Centre for Biotechnology Information (NCBI). 38,259 of the 55,997 transcripts matched to a known protein within the database with a score of E < 10^−6^). 32 % of the transcripts (17,738) did not have a BLAST result. Some of these sequences may not have a homolog in *Drosophila*; others may be from non-coding RNA sequences that are polyadenylated. Some sequences may be unmatched due to assembly errors. We used Blast2GO to assign Gene Ontology (GO) terms to transcripts with BLASTX matches. 34,332 transcripts were assigned GO terms (Fig. 3).

We also carried out an analysis to find out which metabolic processes predominate in the light organ. First of all we mapped all of the Illumina light organ reads back to the full Illumina transcriptome, then used an FDR adjusted enrichment test for the light organ over the whole transcriptome with 10 % as the cutoff (so that transcripts with less than 10 % of their length mapped by fragments were counted as not expressed). We used Blast2GO to assign GO terms to this subset of light organ transcripts. The results showed that there are a large range of metabolic processes occurring in the light organ, although none that could be confidently linked to its bioluminescent function (see Additional file 2: Table S1).

### Read mapping, measurement of gene expression and differential expression analyses

For each experiment we assembled a database of transcripts *de novo*, since there is no reference genome available for *Arachnocampa*.

#### 454

90.78 and 87.41 % of reads from the 454 light organ and non-light organ libraries, respectively, uniquely mapped to transcripts in the 454/CLC Genomics Workbench assembly. We normalized expression values for each sample by scaling so that the median values were made equal. A comparison of normalized expression values provided us with a list of 34 transcripts found at ≥ 10-fold higher levels in the light organ sample than in the rest of the glowworm body sample (Table 4). In addition, 4616 different transcripts were expressed in the light organ sample, but not in the rest of the glowworm body sample. The top 30 of these, ranked according to normalised expression levels, are listed in Table 5.

**Table 4:** Differential expression analysis for 454 data: transcripts expressed ≥ 10-fold more highly in glowworm light organ tissue than in non-light organ tissue

		Light organ			Rest of body					
Contig number	Gene length (bp)	Total gene reads	Normalized expression values	RPKM	Total gene reads	Normalized expression values	RPKM	Difference (normalized values)	Fold change (normalized values)	Putative identity from BLASTX search hits
16656	711	955	2387.2	2876.3	2	7.4	5.9	2379.8	322.6	phosphatidylethanolamine-binding protein
12303	1832	1702	1651.2	1989.5	9	13	10.3	1638.2	127.0	luciferin 4-monooxygenase
10195	478	2588	9622.5	11594.2	14	77.4	61.6	9545.1	124.3	luciferin 4-monooxygenase
13441	1452	13014	15929.3	19193.3	87	158.4	126.0	15770.9	100.6	luciferin 4-monooxygenase
12283	1279	446	619.8	746.7	3	6.2	4.9	613.6	100.0	glutathione s-transferase
4637	1047	273	463.4	558.4	2	5.1	4.0	458.3	90.9	sulfotransferase
12325	1830	2569	2495	3006.2	21	30.3	24.1	2464.7	82.3	luciferin 4-monooxygenase
2377	1303	339	462.4	557.1	4	8.1	6.5	454.3	57.1	aminoacylase-1
2684	2024	65	57.1	68.8	1	1.3	1.0	55.8	43.9	otopetrin
17552	746	513	1222.2	1472.6	8	28.4	22.5	1193.8	43.0	cleavage stimulation factor 64 kDa subunit
11885	2198	49	39.6	47.7	1	1.2	1.0	38.4	33.0	ATP-binding cassette transporter
3008	1206	49	72.2	87.0	1	2.2	1.7	70	32.8	GST-containing flywch zinc-finger protein
16267	1647	42	45.3	54.6	1	1.6	1.3	43.7	28.3	protein lsm14 homolog b-like
12345	961	293	541.9	652.9	7	19.3	15.3	522.6	28.1	carboxylesterase
2668	928	72	137.9	166.1	2	5.7	4.5	132.2	24.2	tpa_inf: hdc07468
12310	1030	452	779.9	939.7	13	33.4	26.5	746.5	23.4	carboxylesterase
4414	2035	138	120.5	145.2	4	5.2	4.1	115.3	23.2	sodium-dependent phosphate transporter
4376	704	32	80.8	97.3	1	3.8	3.0	77	21.3	carbonic anhydrase
11948	1363	29	37.8	45.6	1	1.9	1.5	35.9	19.9	facr2_drome ame: full = fatty acyl- reductase cg8303
16727	1498	30	35.6	42.9	1	1.8	1.4	33.8	19.8	short-chain dehydrogenase
16747	1127	28	44.2	53.2	1	2.3	1.9	41.9	19.2	cofilin actin-depolymerizing factor homolog
3316	1969	21	19	22.8	1	1.3	1.1	17.7	14.6	isoform a
9802	1035	22	37.8	45.5	1	2.6	2.0	35.2	14.5	kynurenine aminotransferase
9665	1782	19	18.9	22.8	1	1.5	1.2	17.4	12.6	transposable element tc3 transposase
11695	1953	56	51	61.4	3	4.1	3.2	46.9	12.4	protein yellow
12223	1142	36	56	67.5	2	4.6	3.7	51.4	12.2	ganglioside-induced differentiation-associated protein 1
10010	267	18	119.8	144.4	1	9.9	7.9	109.9	12.1	hypothetical protein Sulku_2095
3263	1410	17	21.4	25.8	1	1.9	1.5	19.5	11.3	multiple inositol polyphosphate phosphatase
4985	851	16	33.4	40.3	1	3.1	2.5	30.3	10.8	ubiquitin fusion degradaton protein
13509	589	32	96.6	116.3	2	9	7.1	87.6	10.7	kda midgut protein
16372	1493	16	19	22.9	1	1.8	1.4	17.2	10.6	cytochrome p450
4731	700	31	78.7	94.8	2	7.6	6.0	71.1	10.4	isoform b
7053	817	15	32.6	39.3	1	3.2	2.6	29.4	10.2	No significant similarity found
16679	960	45	83.3	100.4	3	8.3	6.6	75	10.0	hypothetical conserved protein

Transcripts are ranked according to normalized expression values; read counts were scaled so that the median values were made equal. RPKM = reads per kilobase of exon model per million mapped reads

**Table 5:** Differential expression analysis for 454 data: top 30 transcripts expressed in glowworm light organ tissue but not in non-light organ tissue

Contig number	Gene length (bp)	Total gene reads	Normalized expression values	RPKM	Putative identity from BLASTX search hits
13443	427	347	1444.3	1740.2	No significant similarity found
10414	304	64	374.2	450.8	luciferin 4-monooxygenase
2392	1330	156	208.5	251.2	heat shock protein 70
13448	565	65	204.5	246.4	sugar transporter sweet1-like isoform 2
13375	816	82	178.6	215.2	No significant similarity found
14967	142	10	125.2	150.8	No significant similarity found
94	215	15	124	149.4	No significant similarity found
860	68	4	104.5	126.0	No significant similarity found
4416	1031	54	93.1	112.2	No significant similarity found
10178	78	4	91.1	109.8	No significant similarity found
10298	119	6	89.6	108.0	hypothetical protein Sulku_2095
14957	504	25	88.2	106.2	No significant similarity found
161	142	7	87.6	105.6	No significant similarity found
17729	168	8	84.6	102.0	No significant similarity found
706	85	4	83.6	100.8	No significant similarity found
10061	152	7	81.8	98.6	troponin i
14955	181	8	78.6	94.6	No significant similarity found
2879	1600	70	77.8	93.7	protein maelstrom homolog
80	70	3	76.2	91.8	No significant similarity found
14930	118	5	75.3	90.7	No significant similarity found
15799	49	2	72.5	87.4	No significant similarity found
10252	74	3	72.1	86.8	No significant similarity found
7300	228	9	70.2	84.5	No significant similarity found
10131	104	4	68.4	82.4	No significant similarity found
1453	55	2	64.6	77.9	No significant similarity found
15071	83	3	64.2	77.4	No significant similarity found
14973	194	7	64.1	77.3	No significant similarity found
9	201	7	61.9	74.6	No significant similarity found
15207	173	6	61.6	74.3	No significant similarity found
788	117	4	60.8	73.2	No significant similarity found

Transcripts areranked according to normalized expression values; read counts were scaled so that the median values were made equal. RPKM = reads per kilobase of exon model per million mapped reads

#### Illumina

We mapped reads for each of the six samples in this experiment onto the Illumina/Trinity reference transcriptome assembly. 91 % of the reads from all six samples were matched to transcripts from the assembly set. Since there were three separate samples for each tissue type, we performed inter-sample normalization, so that cross sample comparison could be carried out without being biased by sequencing depth. We used a TMM method (trimmed-mean of M values) to accommodate the difference in sequencing depth between replicates by finding a scaling factor for each library that minimizes the log-fold changes between the samples. The scaling factor is used to normalise the expression values for each sample.

Differential expression analysis was carried out on transcript expression values from all six Illumina-sequenced samples after adjusting for library size. Only six transcripts were considered to be expressed to a significantly higher level in the light organ than in the non-light organ tissue (false discovery rate of < 0.1); these are listed in Table 6.

**Table 6:** Transcripts from the Illumina sequenced samples that are significantly more highly expressed in the light organ relative to the rest of the body in the glowworm

Transcript number	Rank	Log2 fold change	Log2 of read count per million	*P* value	False discovery rate	Putative identity from BLASTX search hits
64201-seq1	1	9.90	11.88	1.68E-06	0.054	acyl-CoA synthetase/luciferin 4-monooxygenase
62762	2	10.15	15.44	3.05E-06	0.054	acyl-CoA synthetase/luciferin 4-monooxygenase
60014	3	9.91	9.09	3.14E-06	0.054	aminoacylase
51138	4	9.71	10.98	4.75E-06	0.054	phosphatidylethanolamine-binding protein
64201-seq2	5	10.05	11.12	5.13E-06	0.054	acyl-CoA synthetase/luciferin 4-monooxygenase
56768	6	9.81	10.63	5.56E-06	0.054	glutathione S-transferase

A positive value for log2 fold change indicates over-expression in light organ relative to non-light organ tissue

#### Differential expression analysis validation

When comparing the sequences of differentially expressed transcripts in the two experiments, it became apparent that the six transcripts from the Illumina experiment were all found to be in the top eight ranked transcripts from the 454 experiment (Table 7), with one transcript (annotated as 62762 in the Illumina experiment) listed twice in the top eight. The close matching of the results from these two separate experiments, despite differences in samples, sequencing platforms and analytical algorithms, effectively validates these results.

**Table 7:** Common differentially expressed transcripts from 454 and Illumina sequencing analyses

Rank (Illumina)	Transcript number (Illumina)	Rank (454)	Contig number (454)	Protein size (kDa)	Putative identity from BLASTX search hits	Speculative function
1	64201-seq1	7	12325	58.6	acyl-CoA synthetase/luciferin 4-monooxygenase	bioluminescence catalysis
2	62762	3,4	10195, 13441	59.0	acyl-CoA synthetase/luciferin 4-monooxygenase	bioluminescence catalysis
3	60014	8	2377	45.0	aminoacylase	processing of bioluminescent substrate
4	51138	1	16656	20.1	phosphatidylethanolamine-binding protein	ATP binding
5	64201-seq2	2	12303	58.2	acyl-CoA synthetase/luciferin 4-monooxygenase	bioluminescence catalysis
6	56768	5	12283	25.0	glutathione S-transferase	directly or indirectly involving glutathione in bioluminescence

The number of differentially expressed transcripts is small in both analyses. This may be because there are Malpighian tubules in both tissue types: the light organ tissue sample contains the modified Malpighian tubule tips that produce light, and the non-light organ sample contains the remaining non-luminescent parts of the Malpighian tubules. Presuming the modified Malpighian tubule tips retain some of the same functionality as the remainder of the tubules, both samples would share many of the same transcripts.

### Functional annotation of genes highly expressed in the light organ

The proteins encoded by these six transcripts are likely to play important roles in the bioluminescence of the glowworm, assuming that the transcript levels are equivalent to protein levels. In order to find out what these roles might be, we searched for annotated sequence homologs in the publically available non redundant Genbank protein sequence database using the BLASTX algorithm. The resulting annotations will need to be confirmed using biochemical investigation of both the native and recombinant forms of the encoded proteins.

#### *64201-seq1, 64201-seq2, 62762*

**Fig. 4:**
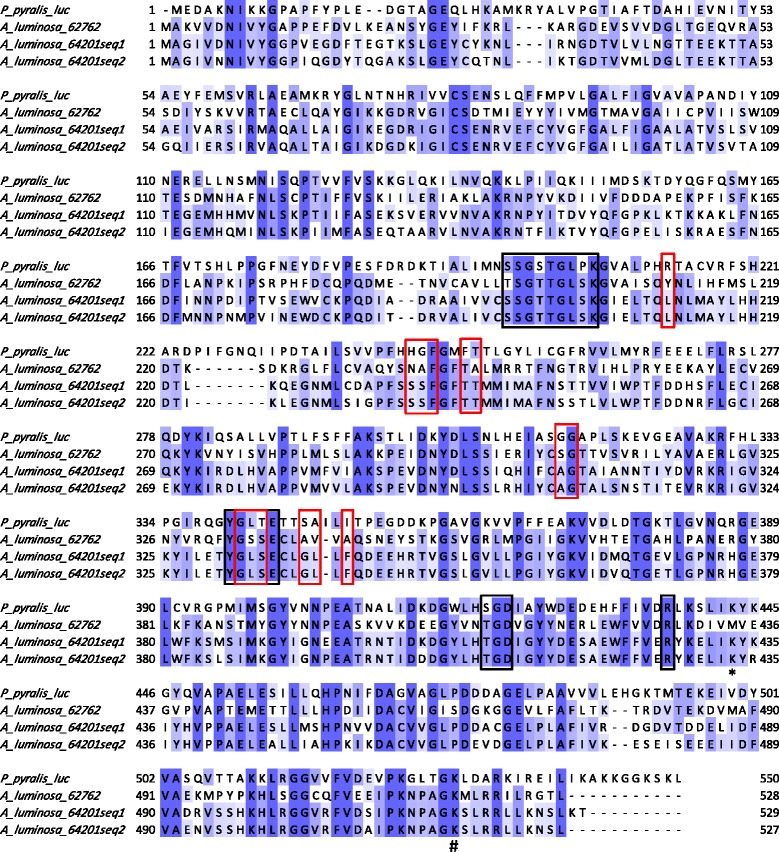
Alignment of amino acid sequences encoded by transcripts 64201-seq1, 64201-seq2, and 62762. The alignment was carried out using Clustal Omega (http://www.ebi.ac.uk) and visualised using Jalview (http://www.jalview.org). Residues are colored according to conservation of sequence identity (dark blue: 100 % conserved). Black boxes represent positions of ATP-binding motifs conserved throughout the ANL superfamily [33], and red boxes represent luciferin-binding residues from the beetle luciferase [62, 63]. The residue marked with a ‘#’ plays a key role in the firefly luciferase adenylation half reaction, and the residue marked with a ‘*’ plays a key role in the oxidation (light-producing) half reaction [64]

The proteins encoded by these three transcripts all display the signature motifs of the ANL superfamily of adenylating enzymes [33] (Fig. 4). The three main subfamilies in the ANL superfamily include the Acyl-CoA synthetases, the NRPS adenylation domains, and the beetle (firefly) Luciferase enzymes. Despite catalyzing a wide range of different overall reactions, ANL enzymes all use a two-step reaction where the first step is always the activation of a carboxylate substrate with ATP to form an adenylate intermediate. These three glowworm proteins are very similar to firefly luciferase (31 to 37 % identical with luciferase from *Photinus pyralis*). The glowworm proteins are very similar and two appear to be isoforms. 64201_seq1 and 64201_seq2 are 79 % identical (the Trinity software labelled them with the same number and ‘seq1’ or ‘seq2’ to reflect this), and 62762 is 43 and 45 % identical to 64201_seq2 and 64201_seq1, respectively. The differences between these three proteins do not appear to be due to alternative splicing, since the differences in sequence are scattered throughout the proteins (Fig. 4).

#### 60014

The protein encoded by this transcript has 44 % amino acid sequence identity with human aminoacylase-1. Amino acids can be stored with an acyl group attached to their N-terminus, which makes them more stable. Aminoacylase-1 removes the acyl group, making the amino acid available for protein synthesis and other metabolic roles [34, 35], and acts specifically on mercapturic acids (S-conjugates of *N-*acetyl-L-cysteine) and neutral aliphatic *N-*acyl-α-amino acids. If the glowworm luciferin is revealed to be a derivative of an amino acid, as it appears to be for the unrelated Siberian luminous earthworm *Fridericia heliota* [36], it is possible that the glowworm might store the luciferin substrate in a stable acylated form and 60014 could deacetylate the substrate, making it available for the bioluminescent reaction. There is, however, no other evidence for this involvement, and the identity of the luciferin is unclear at this stage.

#### 51138

This transcript encodes a member of the phosphatidylethanolamine-binding protein superfamily. Proteins in this family generally play roles in modulating cellular signaling [37]. At a molecular level they have been found to bind nucleotides, opioids and phosphatidylethanolamine. They can also bind kinases, leading to inhibition or activation of signalling pathways. From this information we can infer that 51138 may be involved in the modulation of a bioluminescence signaling pathway.

#### 56768

This protein is a member of the glutathione S-transferase (GST) family of proteins, which play an important role in insecticide resistance and protection against oxidative stress. Members of this family catalyze the conjugation of reduced glutathione to a variety of exogenous and endogenous hydrophobic electrophiles for the purpose of detoxification [38]. 56768 has closest homology with the Delta class of insect GSTs, and has 45 % identity with a mosquito Delta GST that has DDT dehydrochlorinase activity [39]. Therefore glutathione may play a role in glowworm bioluminescence, either directly or indirectly, although it is unclear at this stage what this role might be.

### Evolution of bioluminescence in glowworms

It is interesting that the firefly and glowworm luciferase enzymes belong to the same family of enzymes (assuming that one or more of the three ANL proteins from the light organ are confirmed biochemically to be the glowworm luciferases). In one way it is not unexpected as both are from the same class (Insecta), but because of the evolutionary distance between the glowworm and the firefly, and because the two bioluminescent systems use different substrates [9], it seemed likely that the proteins would differ significantly. However, these differential transcriptomic analyses, and the observation that both the glowworm and the firefly use ATP as a cofactor [10], suggest that the two luciferases may indeed have evolved from the same ancestral non-bioluminescent enzyme. Other explanations for both glowworms and fireflies having a similar luciferase enzyme are unlikely, such as horizontal gene transfer, or the possibility that the insect ancestral to both Coleoptera and Diptera was bioluminescent and passed the bioluminescent gene to both fireflies and glowworms but not to the vast majority of other non-bioluminescent Coleoptera and Diptera that exist today. The independent evolution of the two bioluminescent enzymes from a non-bioluminescent ancestral acyl-CoA synthetase enzyme is most likely, because it has been well established that the beetle luciferases evolved from non-bioluminescent acyl-CoA synthetases [40–42]. In addition, acyl-CoA synthetases from two nonluminous insects, the mealworm *Zophobas morio* [43] and the fruit fly *Drosophila melanogaster* [44], have been shown to bioluminesce faintly in the presence of either the firefly luciferin substrate or a synthetic analog of this luciferin. A firefly luciferase-like gene has also been identified from an animal unrelated to insects: the siliceous sponge *Suberites domuncula* [45]; however, this claim needs to be confirmed [46], especially since the native luciferase protein itself has not yet been isolated from the sponge tissue.

**Fig. 5:**
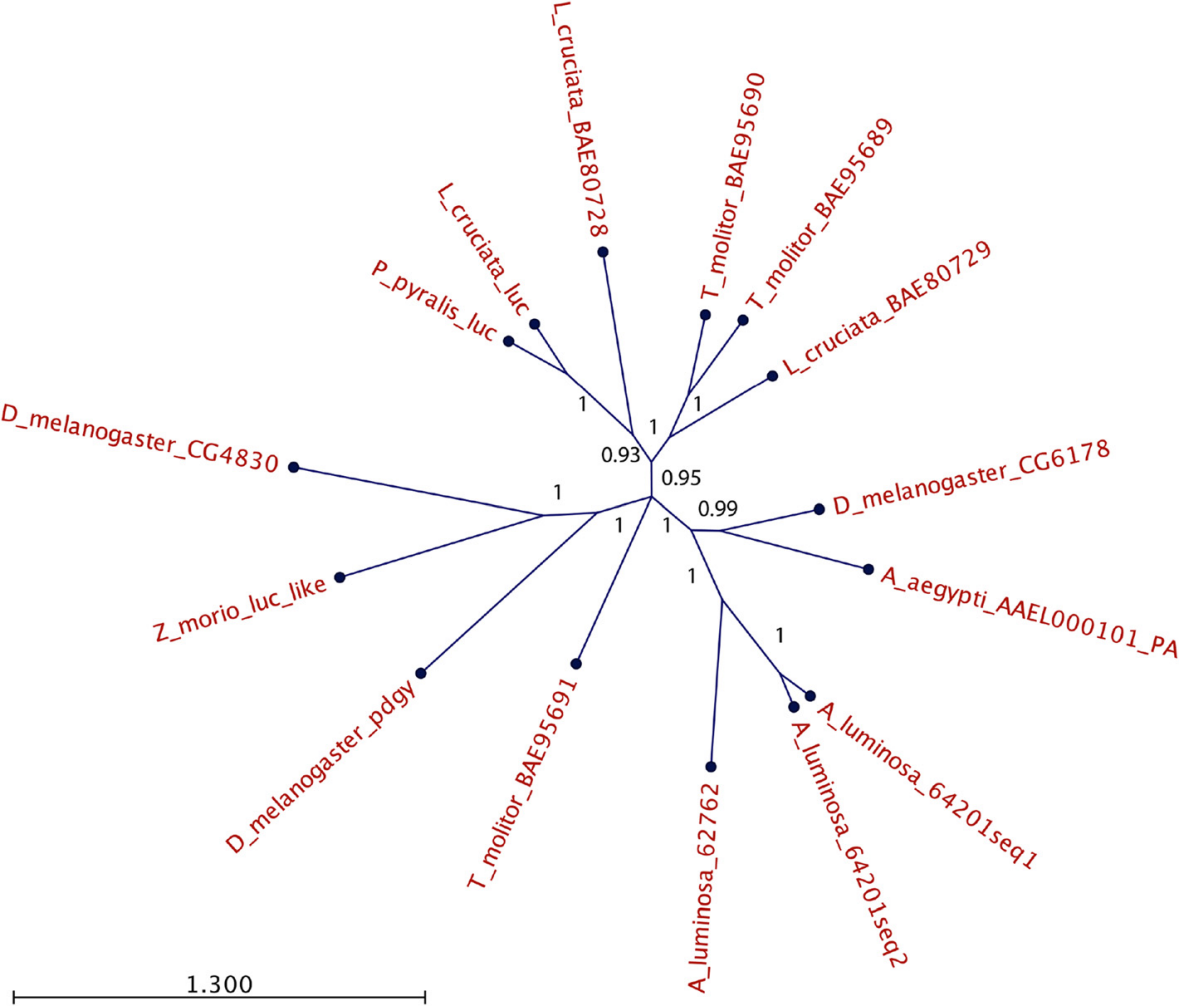
Unrooted phylogenetic tree of luciferases and related proteins. Details for each protein are provided in Table 8

We carried out a phylogenetic analysis of the three glowworm luciferase-like proteins along with known firefly luciferases and other luciferase-like proteins from various insects. Notably, the three glowworm proteins are grouped together and are placed closer to proteins from non-bioluminescent dipteran insects (*D. melanogaster and A. aegypti*) than to firefly luciferases (see Fig. 5 and Table 8).

**Table 8:** Details of luciferases and related proteins included in the phylogenetic analysis (Fig. 5)

Organism	Name/accession number of protein	Function	Catalyses bioluminescence with firefly luciferin?	Reference
*Arachnocampa luminosa* (glowworm)	64201_seq1	candidate luciferase	Not tested	-
*Arachnocampa luminosa* (glowworm)	64201_seq2	candidate luciferase	Not tested	-
*Arachnocampa luminosa* (glowworm)	62762	candidate luciferase	Not tested	-
*Photinus pyralis* (North American firefly)	luciferase/P08659	luciferase	Yes	[14]
*Luciola cruciata* (Japanese firefly)	luciferase/P13129-1	luciferase	Yes	[14]
Luciola cruciata (Japanese firefly)	LcLL1/BAE80728	luciferase-like protein	No	[42, 65]
Luciola cruciata (Japanese firefly)	LcLL2/BAE80729	luciferase-like protein	No	[42, 65]
*Drosophila melanogaster* (fruit fly)	CG6178	firefly luciferase homolog (closest homolog to firefly luciferase in *D. melanogaster*)	No	[65]
*Drosophila melanogaster* (fruit fly)	pdgy/NP_572988	acyl Co-A synthetase	Not tested	[66]
*Drosophila melanogaster* (fruit fly)	CG4830	predicted acyl-CoA synthetase	No	[65]
*Aedes aegypti* (mosquito)	AAEL000101-PA	AMP-dependent CoA ligase homolog	Not tested	-
*Tenebrio molitor* (mealworm)	TmLL-1/BAE95689	luciferase-like protein	No	[67]
*Tenebrio molitor* (mealworm)	BAE95690/TM-LL2	acyl-CoA synthase	No	[67]
*Tenebrio molitor* (mealworm)	TmLL-3/BAE95691	luciferase-like protein	No	[67]
*Zophobas morio* (giant mealworm)	Luc-like	luciferase-like protein	Weak bioluminescence	[43]

It is uncertain why there are three isoforms of the firefly-luciferase-like protein expressed in the glowworm light organ. The firefly *Luciola cruciata* expresses three isoforms, where only one has bioluminescent activity [42], so it is possible that the glowworm may also follow a similar pattern of gene duplication. Gene duplication followed by enzyme diversification and the development of novel functions has been a feature previously observed in vertebrate acyl-CoA synthetase genes [47]. There appear to be many duplication events in the evolution of the acyl-CoA synthetase enzyme family in insects. We used tBLASTn to search the total glowworm transcriptome from the Illumina experiment and found that the three glowworm proteins have numerous paralogs: between 65 and 68 transcripts have 20 to 50 % identity with the three protein sequences. In addition, the limited transcriptional profiling studies by Silva *et al.* mentioned previously found about 1 % of the 537 *A. luminosa* light organ cDNAs and 2 % of the 572 firefly lantern cDNAs sequenced were members of the acyl-CoA synthetase enzyme family (also known as AMP/CoA-ligases) [29, 30].

## Conclusions

This report describes the first high-throughput transcriptome sequencing of the New Zealand glowworm, and the use of comparative RNAseq to identify genes expressed in luminescent tissue that are involved in bioluminescence. Two separate differential expression analyses have identified six genes that are significantly more highly expressed in the light organ than in non-luminescent tissue. These genes encode putative aminoacylase, GST and phosphatidylethanolamine-binding proteins, and, most notably, three proteins that are homologs of firefly luciferase, at least one of which we expect to be the glowworm luciferase.

Interestingly, in the Silva *et al.* study of glowworm light organ cDNAs [29], one of the members of the acyl-CoA synthetase enzyme family sequenced showed 44–45 % identity with railroad worm luciferases, and 2.1 % of the transcripts sequenced were putative GST proteins. This, combined with our research underlines the potential importance of these sequences in glowworm bioluminescence.

Further biochemical studies are required to confirm that one or more of the candidate luciferases are able to produce light. These studies should include two approaches: firstly, produce these proteins in a recombinant form and assay them for bioluminescent activity using the native luciferin substrate extracted from the glowworm; secondly, isolate the native luciferase protein(s) from the light organ tissue, using the same assay to track bioluminescent activity, and identify the isolated protein(s) using mass spectrometry and the transcriptome database generated in the current study. If the candidate luciferase(s) is/are confirmed, then this will show that this enzyme has independently evolved the ability to produce light at least twice in extant organisms, in New Zealand glowworms and in fireflies, but with different substrates. Once the identity of the glowworm enzyme has been confirmed, and the chemistry of the glowworm substrate has been revealed, potential applications of the novel glowworm bioluminescence system can be explored.

## Methods

### Sample collection and RNA extraction

*A. luminosa* is an endemic species and is of particular interest to the indigenous Maori people of New Zealand. Therefore local Maori were consulted about this research through Te Komiti Rakahau ki Kāi Tahu (the Ngāi Tahu Research Consultation Committee). As glowworms are invertebrates, the approval of an ethics committee was not required. *A. luminosa* is not protected, and no experiment was performed on living animals.

*A. luminosa* specimens were collected from locations around New Zealand, including Dunedin, Te Anau and Palmerston North. Live glowworms were snap frozen by being placed on foil above dry ice, and then stored until required at −80 °C. Using a razor, light organs were removed from the glowworm bodies while still frozen. Light organ samples contained only white light organ tissue, and non-light organ samples contained the rest of the glowworm body and head (darker tissue) with white light organ tissue removed entirely. Tissues were homogenised with TRIzol® Reagent (Invitrogen) and a glass dounce homogeniser; total RNA was extracted using UltraPure™ (Phenol: Chloroform:Isoamyl Alcohol; Invitrogen), and then further purified using the RNeasy Kit (Qiagen). RNA quantification and integrity assessment were performed for each sample on an RNA chip (Bioanalyzer 2100, Agilent Technologies).

### cDNA library construction, sequencing and quality control

#### 454

Two hundred μg of non-light organ RNA (prepared from ~200 mg of non-light organ tissue from eight glowworms) and 65 μg of light organ RNA (prepared from ~420 mg of light organ tissue from 172 glowworms) were sent to the 454 Life Sciences Sequencing Center (Roche, Branford, Connecticut, USA) for mRNA enrichment, cDNA library construction (including mRNA fragmentation using a zinc chloride solution, cDNA synthesis using random hexamer primers and adaptor ligation) and subsequent 454 sequencing on a Roche GS FLX Titanium Genome Sequencer. Each sample was sequenced on one half of a picotiter plate. Low quality reads were discarded (quality limit of 0.05). We also removed: ambigious nucleotides (maximum of 5 nucleotides allowed), terminal nucleotides (1 each from both the 5′ and 3′ ends), adapter sequences and sequences less than 20 nucleotides in length.

#### Illumina

Light organ and non-light organ total RNA was prepared separately from three individual glowworms (0.6 to 1.2 μg of RNA for each light organ sample, and 8.8 to 27.0 μg of RNA for each non-light organ sample). The six samples, each with an RNA Integrity Number (RIN) of over 6, were sent to the Otago Genomics and Bioinformatics Facility, where mRNA was isolated using oligo-dT magnetic beads, and cDNA libraries were constructed using the Illumina TruSeq Stranded mRNA Sample Preparation Kit. Sequencing was carried out on an Illumina HiSeq-2000 sequencer, generating 100 bp paired-end reads. Each sample was run on one eighth of a sequencing lane. Adaptor sequences were trimmed from reads using fastq-mcf [48], and bases with low quality phred scores trimmed (cut-off phred score of Q20). Adapter and quality trimmed reads less than 50 nucleotides in length were discarded using the SolexaQA package [49]. Reads were quality assessed using FASTQC (http://www.bioinformatics.bbsrc.ac.uk/projects/fastqc).

### *De novo* assembly

#### 454

We used the CLC Genomics Workbench v5.1 (http://www.clcbio.com) to *de novo*-assemble 454 reads from the combined light organ and non-light organ samples into a single, non-redundant contig dataset. Singletons were merged into the contig set. Assembly parameters were set at the program default settings.

#### Illumina

Reads combined from all light organ and non-light organ samples were assembled using the Trinity software package [50]. Assembly parameters were adjusted for Illumina stranded paired end sequencing (i.e. –left and –right for both R1 and R2 using --SS_lib_type RF for the stranded library type).

### Read mapping and measurement of gene expression

#### 454

We used the CLC Genomics Workbench to map reads from each of the 454 light organ and non-light organ samples back onto the CLC Genomics Workbench/454 assembly dataset. Expression of genes was represented by the abundance of reads uniquely mapped to each contig in the assembly. Abundance was expressed as RPKM (reads per kilobase of exon model per million mapped reads) or normalized expression values (the contig read counts were scaled so that the median values were made equal).

#### Illumina

For each of the six Illumina samples, reads were mapped onto the Trinity/Illumina assembly using Bowtie 2 [51], and transcript abundance for each sample was estimated using the RSEM package [52]. Per sample relative abundance was estimated using FPKM (fragments per kilobase of transcript per million fragments mapped), EC (expected counts) and TPM (transcripts per million). For cross-sample comparisons, we normalized raw read counts for these samples using the TMM method (trimmed-mean of M values; a weighted trimmed mean of the log expression ratios [53]) as implemented in the *edgeR* package [54] (http://www.bioconductor.org).

### Differential expression analyses

To identify candidate bioluminescence-related genes from our datasets, we compared normalized transcript abundance values between corresponding light organ and non-light organ samples.

#### 454

In order to detect the differentially expressed genes between the two 454-sequenced samples, a two-group differential analysis was performed in CLC Genomics Workbench on normalized expression values.

#### Illumina

Genes with significantly different levels of expression between light organ and non-light organ samples (a single factor design) were identified using the quantile-adjusted Conditional Maximum Likelihood method (qCML) in the *edgeR* package [55]. We considered transcripts to be differentially expressed to a significant level at a false discovery rate at < 0.1.

### Functional annotation

Transcripts that were expressed at significantly higher levels in light organ compared with non-light organ tissue were annotated by searching the GenBank non-redundant database at the NCBI (http://www.ncbi.nlm.nih.gov) using the BLASTX software [56] with default parameters. Automated annotation of the transcriptome database was carried out using BLASTX searches to the NCBI non-redundant database within the Blast2GO program v2.8.0 (http://www.blast2go.com) [57]. An E-value cut-off of 1E^−6^ was used to identify similar annotated proteins from which function could be inferred.

### Phylogenetic analysis

Multiple alignments were produced using MUSCLE [58, 59]. A phylogenetic analysis was carried out in MrBayes [60], using the WAG model of protein evolution [61] which was found to be the most appropriate after tests with mixed models. Monte-Carlo Markov chains were run for 1 000 000 generations with the initial 25 % of trees discarded as burn-in. The consensus trees produced were visualized with CLC Genomics Workbench.

### Availability of supporting data

The raw sequence data from the Illumina experiment was submitted in FASTQ format to the NCBI Sequence Read Archive (SRA) database (accessions: SRR2241413, SRR2283829, SRR2283830, SRR2283831, SRR2283975 and SRR2284246) and are also accessible through the BioProject accession PRJNA290397 (http://www.ncbi.nlm.nih.gov/bioproject/290397). The Illumina/Trinity transcriptome assembly has been deposited at the NCBI Transcriptome Shotgun Assembly (TSA) database and is available at the DNA DataBank of Japan (DDBJ), the European Molecular Biology Laboratory (EMBL), and GenBank at NCBI under the accession GDQV00000000.

## Additional files

Additional file 1: Figure S1. Workflows for the two differential expression analysis experiments (PDF 44 kb) Additional file 2: Table S1. Gene Ontology terms of transcripts found in the glowworm light organ (XLSX 231 kb)

## Abbreviation

RPKM: Reads per kilobase per million
