# Supplementary figures and images for: Comparative RNA seq analysis of the New Zealand glowworm Arachnocampa luminosa reveals bioluminescence-related genes
Source: BMC Genomics. 2015 Oct 21;16:825. doi: 10.1186/s12864-015-2006-2 (PMC4617951; doi:10.1186/s12864-015-2006-2)

Supplementary Figure 1:  
Workflows for the two differential expression analysis experiments

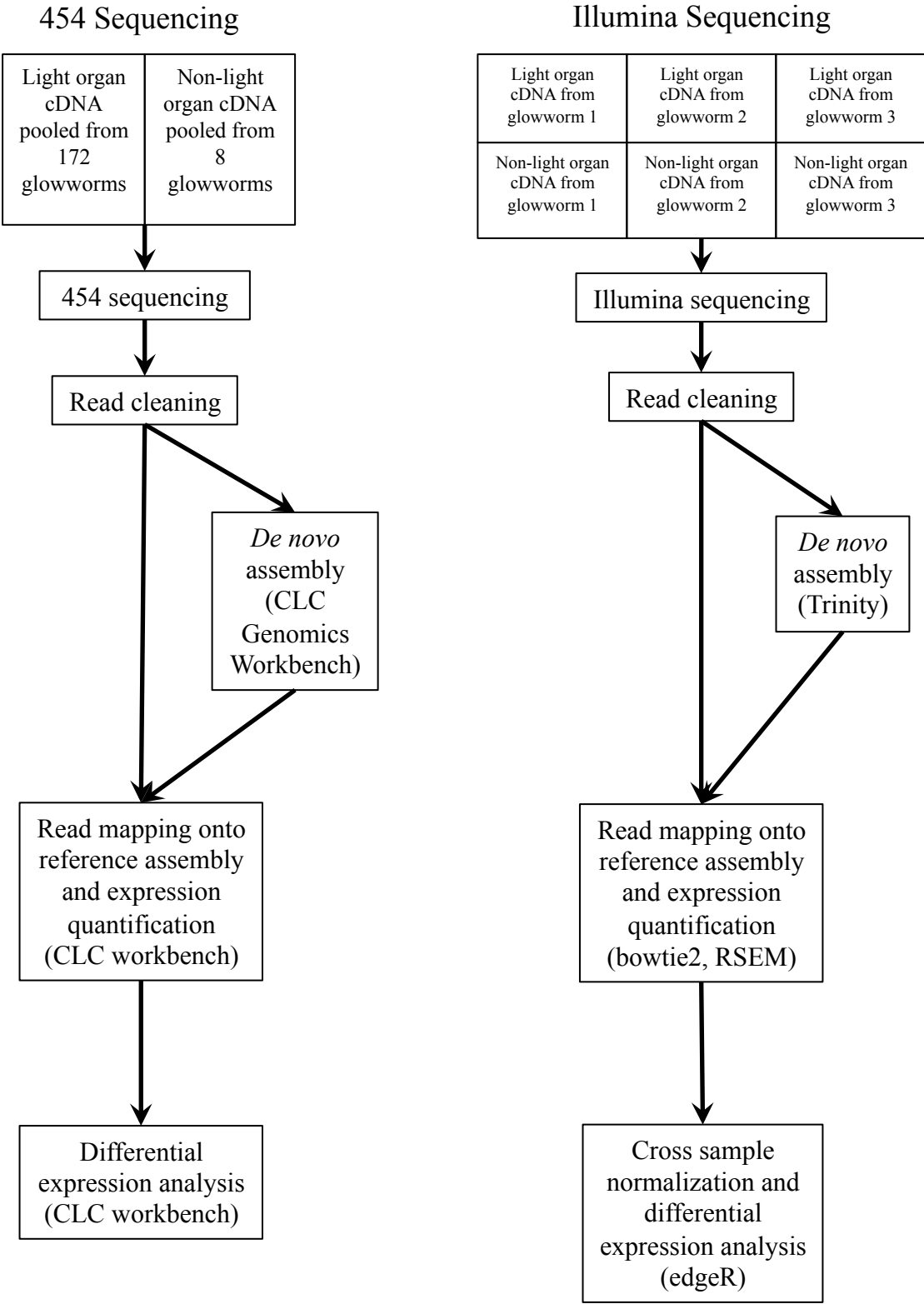

Supplement: Additional file 1: Figure S1. — Workflows for the two differential expression analysis experiments (PDF 44 kb) [file 12864_2015_2006_MOESM1_ESM.pdf]
